# How do people with neurofibromatosis type 1 (the forgotten victims) live? A grounded theory study

**DOI:** 10.1111/hex.13413

**Published:** 2022-01-19

**Authors:** Samira Foji, Eesa Mohammadi, Akram Sanagoo, Leila Jouybari

**Affiliations:** ^1^ School of Nursing and Midwifery Sabzevar University of Medical Sciences Sabzevar Iran; ^2^ Department of Nursing, School of Nursing and Midwifery Golestan University of Medical Sciences Gorgan Iran; ^3^ Department of Nursing, Faculty of Medical Sciences Tarbiat Modares University Tehran Iran; ^4^ Nursing Research Center Golestan University of Medical Sciences Gorgan Iran

**Keywords:** grounded theory, life, neurofibromatosis, qualitative study, rare disease

## Abstract

**Background:**

Neurofibromatosis type I (NF1) is a rare genetic disorder, associated with some physical symptoms including spots and tiny bumps on the skin, and internal organ involvement. People suffering from neurofibromatosis face various challenges in their daily lives. However, there is little understanding on how patients deal with neurofibromatosis. This study aimed to investigate the life challenges of patients with NF1.

**Methods:**

This qualitative study was performed by implementing a grounded theory with the cooperation of the Society for Neurofibromatosis Patients over the course of 15 months in 2019 across 4 provinces in Iran. Twenty‐four patients with NF1 were interviewed. An analysis was performed using the constant comparative method.

**Findings:**

The results of the analyses indicated that the major concern of the NF1 patients was feelings of failure and falling behind in life. In the face of failure in life in such a context, patients used the main strategy of “unsuccessful struggle to escape” the disease and its complications, which was represented itself in the forms of ‘hopelessness and impatience’, ‘suicidal thoughts and unsuccessful suicide attempts’, ‘isolation and seclusion’, ‘expressing complaints and grievances to God’, ‘hiding the disease’ and ‘hopelessness and refusing to receive care’. The implementation of such strategies helped patients reduce tension and achieve a temporary, though vulnerable and fragile, sense of relief and peace.

**Conclusion:**

Given an unfavourable life condition, NF1 patients turned to a harmful passive strategy in the face of the challenges posed by the disease.

**Patient or Public Contribution:**

Public contributors were active partners throughout, and co‐authored the paper.

## 1 BACKGROUND

Neurofibromatosis type I (NF1), also known as Von Recklinghausen's disease, is an autosomal dominant disorder affecting the skin and the nervous system. There are no gender, racial or ethnic differences in terms of the occurrence of NF1.^1^ NF1 is the most common type of neurofibromatosis, and affects 1 in 3500 people.^2^, ^3^ The exact prevalence of this disease has not been determined in Iran. Therefore, it is assumed that the prevalence rate of this genetic disorder in Iran is similar to the global prevalence of NF1.^4^ The primary symptoms associated with NF1 include dermatologic complications in the form of ‘café au lait’ spots and patches.^4^, ^5^ NF1 can be associated with potential unpredictable and life‐threatening clinical presentations.^6^ The cosmetic complications associated with neurofibromatosis and the resulting social isolation are the main factors for the depression that the patients experience. It has been found that depression is quite common among patients suffering from NF1. The findings of previous studies have shown that the prevalences of depression and other psychiatric complications were higher in patients with NF1 compared to healthy adults.^7^ Various physical, cognitive and social complications of NF1 can significantly reduce the quality of life of patients.^8^

Knowledge of the personal experiences of patients suffering from NF1 is important for understanding their physical and mental (psychological) health and the social challenges that they face. However, there has been a dearth of qualitative studies on the negative effects of NF1 on the lives and the mental health of adult patients. An impaired social network, concern over the unpredictability of the disease and concerns and worries in relation to cosmetic deformities have been previously reported as challenges experienced by patients with NF1.^9^, ^10^, ^11^

The findings of previous studies conducted in other countries may not be helpful in understanding the experiences of NF1 patients in Iran as these experiences are affected by the geographical location, economic status, cultural background and lifestyle. Hence, the present study was designed to investigate the challenges in life faced by Iranian patients with NF1.

## 2 METHODS

This qualitative study was based on a naturalistic research paradigm using the grounded theory.

### 2.1 Participants

Twenty four adult male and female patients with NF1 from four different provinces of Iran participated in this qualitative study (Table 1). The inclusion criteria were as follows: (1) age older than 18 years, (2) confirmed diagnosis of NF1 according to the Society of Neurofibromatosis, (3) awareness of having the disease, (4) disease history of at least 6 months and (5) willingness to participate in the study by signing a formal consent form. As NF1 is a rare disease, the patients were introduced to the researcher by the Iranian Society of Neurofibromatosis. Purposeful sampling was implemented first, and preliminary interviews were conducted to formulate the primary concepts (themes) and hypotheses. The researcher then followed a theoretical sampling procedure to conduct further interviews with participants, and expand on the emerging concepts and diverging views. Interviews and data collection continued until theoretical data saturation was reached.

**Table 1:** Demographic data of the 24 participants with neurofibromatosis

Code	Age	Sex	Occupation	Marital status	Education level
1	42	Female	Housewife	Single	Illiterate
2	41	Female	Housewife	Married	Primary school
3	38	Female	Kindergarten manager	Single	Master's degree
4	27	Female	Student	Single	Master's degree
5	21	Female	Housewife	Single	High school diploma
6	35	Female	Housewife	Single	High school diploma
7	35	Male	Self‐employed	Divorced	High school diploma
8	52	Male	Unemployed	Single	Primary school
9	26	Male	Unemployed	Single	High school
10	27	Male	Student	Single	Master's degree
11	34	Male	Agency driver	Single	Master's degree
12	37	Female	Housewife	Married	High school
13	33	Female	Housewife	Single	High school diploma
14	25	Female	Dressmaker	Single	Bachelor
15	48	Female	Housewife	Married	High school diploma
16	42	Female	Housewife	Single	Associate degree
17	43	Male	Power department employee	Single	Associate degree
18	34	Male	Municipality employee	Married	High school diploma
19	44	Female	Cleaner	Divorced	Junior high school^a^
20	41	Female	Teacher	Single	Bachelor
21	43	Male	Electrical technician	Single	Bachelor
22	38	Female	Seller	Single	High school diploma
23	36	Female	Housewife	Divorced	Junior high school
24	48	Female	Teacher	Single	Bachelor

*Note*: High school involves 3 years of schooling.

^a^ Middle school is an educational stage, (preparatory stage) in Iran, that lasts for three years.

### 2.2 Interview procedure

Semi‐structured and unstructured interviews were conducted from September 2019 to May 2020. All interviews were audio‐recorded. An unstructured interview format was implemented for the first five interviews with open‐ended questions including ‘tell me about your life experiences’ and ‘what kind of effects has this disease had on your life?’ After obtaining patient responses, their reactions in different situations were recorded and checked. Interviews consisted of probing questions, such as ‘can you explain more about this event?’, ‘why did you show such a reaction?’, ‘what factors caused your reaction?’ and ‘what was the result of your reaction?’ The data from the primary interviews were analysed to formulate the primary concepts. These concepts were used in the subsequent semi‐structured interviews. Overall, 28 interviews were conducted with the 24 participants. Four participants were interviewed twice. The interview duration ranged between 45 and 110 min, with an average duration of 63 min. Theoretical saturation was achieved after the 24th interview. Finally, four more interviews were conducted to ensure that no new data could be obtained on each of the primary concepts.

### 2.3 Data analysis

Analysis was performed using the constant comparative approach proposed by Corbin and Strauss.^12^ The five steps of the grounded theory were used for data analysis. These steps include open coding, developing concepts in terms of their properties and dimensions, context analysis, process analysis and integrating categories. In addition, to fulfil the 10 criteria proposed by Corbin and Strauss,^12^ the researcher used the techniques based on the four criteria of Lincoln and Guba to confirm and ensure the accuracy and strength of the study findings.^12^, ^13^ Max QDA software was used for data analysis.

## 3 RESULTS

The analysis of the collected data from the first and second steps yielded 1492 codes in context, 57 subcategories and 10 categories or main concepts (themes). A thorough examination of these concepts and findings indicated that the main concern of the patients was ‘failure and falling behind in their lives’.

### 3.1 Main concern: Failure and falling behind in life

The concept ‘failure and falling behind in life’ was found to be the main concern and challenge that NF1 patients faced. This concept was defined as a disturbing, uncomfortable feeling experienced by patients that was accompanied by the three feelings of ‘marriage failure’, ‘school failure’ and ‘failure at work’.

Patients with NF1 had the feeling of falling behind in life to the extent that they could not progress normally through the different stages of life. Participants suggested that having NF1 frequently resulted in the feeling of failure in life. A 34‐year‐old man believed that the negative effect of the disease was so extensive that it could change his fate and future. In this respect, he stated that:

*Neurofibromatosis is like a minus sign to the left of the parentheses. It negatively influences whatever comes in the parentheses. I mean, this disease changes the fate of the patient*. (Participant 1)

NF1 patients faced serious challenges when it came to marriage. A variety of factors were reported to negatively influence marriage. Thus, NF1 patients usually experienced failed marriages.

In terms of marriage, a 26‐year‐old male patient in the present study stated that:

*Nobody wants to marry an NF1 patient. Even if one starts a relationship with an NF1 patient, as soon as he or she comes to know about the disease, he or she will definitely reject the marriage proposal*. (Participant 9)

When NF1 patients pursued an employment opportunity, they felt that employers generally refused to give them a job position due to changes in their appearance. As appearance was regarded as an important factor for employers in their decision‐making while processing job applications. Due to their appearance, NF1 patients reported that they mostly faced a negative outcome when they applied for a job. In this respect, one of the participants stated that:

*The other day I went to some place to apply for a secretarial job position. The moment the employer saw my appearance, pointing with her hand to my face, she said, what this look is, your appearance makes our customers feel anxious*. (Participant 19)

One of the major challenges faced by NF1 patients was learning disabilities. On average, 50% of NF1 patients had learning disabilities and difficulties, which resulted in low school achievement and dropout from school over time. One of the participants stated that:

*I am thankful for the bonus scores my teachers gave me, I always got zero in all school subjects. To be honest, I couldn't study. It was over my head. For five consecutive years I studied the first grade primary school. But each time, I was failed and prevented from going to the second grade. Because of all the bad scores I got at school, all the school kids kept their distance from me. Since I was a bad student with the lowest scores, they [other students] didn't make friends with me. Although I spent a lot of time studying, I did not learn anything. I am still the same way. I go through some material over and over again, but after a few minutes I totally forget it, I can't remember anything*. (Participant 15)

### 3.2 Conditions

Data analysis revealed that various factors contributed to the conditions and context that force NF1 patients to focus on their failure and falling behind in life including ‘having an unpleasant appearance due to the spots and patches caused by the disease’, ‘being unable to have kids because of the genetic transferability of the disease’, ‘learning disabilities’ and ‘having limitations in performing daily activities’ (at the individual level), as well as ‘social rejection’, ‘facing aggression from others’, ‘not recognizing social support’ (at the social level) and finally ‘no effective treatment’ (at the national level).

Patients suffering from NF1 claimed that they experienced a sense of failure in diverse situations including marriage and employment due to their unusual appearance.

Another participant, a young girl, stated that her marriage proposal was rejected due to the spots and patches that she had on her body and face:

*The very first time they came over to see me and my family in person, my husband to be and his family brought a man with them. So I kept my Islamic hijab, letting them see my hands and face. After many hours of chatting and talking, they had a positive impression of me and my family. I liked the boy, too. He was hard working, with religious commitment which I valued a lot. They promised to see us again. Two days later, they called us and asked about the spots and patches I have on my skin. They wanted to know how serious they are. I told them that my whole body is covered with spots. They never called us nor did they ask us for an explanation for the spots on my skin*. (Participant 20)

Another important contextual factor causing the feelings of failure and sense of falling behind in life in NF1 patients was their inability to have kids due to the risk of genetic transferability of the disease. In terms of this aspect, one of the participants who had chosen not to get married due to this issue stated that:

*Why should I keep someone next to me who may be forced into a lot of unwanted challenges in her future life? After six months or a year, my wife may decide to have a child as one of her aims in life. Why should I let someone come to live with me, and in this way, impose a lot of limitations on her?* (Participants 10)

Another contextual factor leading to a sense of failure and of falling behind in life was learning disabilities, which were experienced by most NF1 patients. Learning disabilities were considered to be the most important reason for low academic achievement and school dropouts.

Talking about her experiences during her university studies, a 38‐year‐old female participant mentioned the deficits and academic learning difficulties she faced during her university studies, stating that:

*You know, at university I couldn't learn effectively. I even didn't know effective learning skills. I was so slow in learning. Now, I see it was one of the negative side effects of Neurofibromatosis*. (Participant 3)

NF1 patients generally faced physical limitations due to the diverse complications caused by the disease, including skeletal disorders and tumours all over the body. Physical activity limitations in NF1 patients prevented them from performing intense or even normal physical activities, which negatively influenced their performance at school and work.

A 23‐year‐old woman talked about her experiences:

*…I even have difficulty cleaning up my house using a broom or even a vacuum cleaner. Whenever I want to sweep the house, my sisters come over to help me. They do the sweeping. When I do the sweeping myself, I get exhausted. You know I cannot pull the vacuum cleaner around the house. And when it sucks in the carpet, I do not have the strength to separate it from the carpet*. (Participant 4)

NF1 patients believed that they were deprived of social interactions due to social rejection. They felt that this experience could result in the feeling of failure in life.

A 44‐year‐old female participant mentioned her low school achievement and her dropping out of school primarily due to the social rejection that she experienced during her school days. She stated that:

*During my school years, all the kids ran away from me, keeping their distance. This made me see school as a cage that hurt me psychologically. Instead of going to school, I used to go into our garden and hide somewhere. When I came home in the afternoon, I told my mother I had been at school. When I was at school, I was not allowed to be in any game, you know, no one wanted me to be his or her partner or teammate. I was alone and felt lonely all the time. This caused me to have low school achievement, and to run away from school*. (Participant 19)

Generally speaking, NF1 patients faced misconduct from others, including curiosity and questioning, mocking, insults, and disrespect.

A 26‐year‐old male participant talked about how others looked down on him in public places, including swimming pools:

*Once, I remember, I went to the swimming pool. I stood at the corner watching others jumping into the pool because they did not allow me to go into the water. They said ‘if you go into the water, you'll make the water dirty’. I cannot go to the swimming pool because I have spots on my body. I cannot go to the gym because I have spots all over my body. They keep away from me because they think my disease is contagious. They said ‘Look at his body, he is like a spotted dog’*. (Participant 9)

Neurofibromatosis is considered a genetic disorder with no effective cure, and its course is unpredictable. Hence, patients were always waiting for complications and problems.

Another participant stated that she was deprived of a normal life and felt a great deal of psychological distress because NF1 was incurable.

*What bothers me most is the fact that it is not curable. There is even some medical cure for many deadly diseases such as HIV, and cancer. Why shouldn't there exist an effective treatment and cure for our disease? Why should we go through so much suffering, and be deprived of a normal life?* (Participant 15)

### 3.3 Processes

In response to the feeling of failure and falling behind in life (main concern) in the context mentioned above, patients with NF1 commonly used the following strategies: ‘being impatient and hopeless’, ‘thinking of suicide, and unsuccessful suicide attempts’, ‘isolation and seclusion’, ‘complaining to God’, ‘covering and hiding their condition’ and refusing to receive care.

The reactions that the patients showed to the widespread negative impacts of NF1 on different aspects of their personal life were feelings of hopelessness and inability to cope with the disease. Due to feelings of hopelessness and the inability to cope with the disorder, a middle‐aged female participant divorced her husband. She stated that her divorce was in response to the pressure and stress she went through because of her inability to pay for the cost‐of‐living expenses. She stated that:

*I am fed up, I don't want to go on with this life, I am willing and ready to die because I cannot work. I cannot pay the rent all on my own. I cannot pay for the costs of life, food, or clothes. I am hopelessly fighting through life. I feel like I am fighting. I cannot go on alone. That is why I made up my mind to ask for Voluntary euthanasia*. (Participant 23)

Due to the difficult conditions and complications faced by patients with NF1, in addition to the feelings of hopelessness and impatience, these patients sometimes experienced suicidal thoughts and attempted suicide as a strategy to deal with the feelings of failure and falling behind in life. A 26‐year‐old male participant stated that:

*I can't continue anymore. I cannot tolerate the conditions and complications of this disease. It is so hard for me. I am thinking of committing suicide all the time. If I kill myself, I will get relief*. (Participant 9)

Isolation and separation were two other strategies that the NF1 patients used to deal with the feelings of failure and falling behind in life. A 35‐year‐old male participant talked about his isolation and separation from society and staying at home:

*This disease made me feel inferior to others. I escape from the society, or I pass my time passively at home alone. For entertainment, I have just two options, watching TV or sleeping. I don't want to be in the society*. (Participant 7)

Once patients became aware of their disorder and its widespread negative impacts on the different aspects of their lives, including marriage, employment and education, they felt that they did not deserve to have this incurable disease. As a result, the dominant strategy that they used to cope with the feelings of failure and falling behind in life was to complain and express their grievance.

In this respect, a 44‐year‐old woman stated that:

*I complained to God. I asked, ‘For what sin should I go through this suffering?’ I do not complain to people (your creatures), I am asking you, ‘Why have you chosen me to suffer from this disease?’ My constant job is dealing with this disease. You made me lonely. I experience enormous pain and suffering, still I have to work to pay for the treatment costs. I am constantly complaining to God*. (Participant 19)

NF1 patients tried to hide their symptoms and complications in different ways to avoid social stigma and the consequences, including unfair and wrong judgements of people of their genetic disorder.

In this respect, a 42‐year‐old single participant reported that she wore more clothes to hide her complications:

*When you get together, you cannot wear a comfortable clothe (weeping). Currently, a couple of friends and I hold parties regularly. Whenever I am in the party, I wear a scarf. My friends ask me ‘Do you have problems with your hair?’ ‘Why are you wearing a scarf?’ You know, I wear a scarf to cover (hide) the spots and tumors all over my head*. (Participant 16)

When patients with NF1 became aware of the fact that their condition is incurable, they discontinued treatment and other medical care plans.

*I went to see my doctor. The doctor told me honestly that there is no effective treatment for my condition. The doctor told me, ‘Don't waste your money in vain. There is no treatment for this disease. As your stress and anxiety go up, your symptoms and complications become more severe. You need to avoid eating fast foods such as sausages, bologna, and other delicacies’. But I still eat them*. (Participant 12)

### 3.4 Theoretical explanation

Feelings of failure and falling behind in life were the main concerns and worries expressed by the NF1 patients. The conditions and factors faced by NF1 patients included ‘unpleasant appearance due to the spots and tumors’, ‘inability to have kids due to the genetic transferability of the disease’, ‘learning disabilities’, ‘limitations in doing daily life activities’, ‘social rejection and isolation’, ‘facing aggression form others’, ‘perception of no social support’ and ‘incurability of the disease’. Due to these conditions, feelings of failure and falling behind in life developed among NF1 patients.

Faced with the feelings of failure and falling behind in life, NF1 patients were forced to use some ineffective approaches, including hiding their disease from others, seeking isolation and separation, complaining to God, feeling impatient and hopeless and refusing to receive care. In some cases, they contemplated suicide, and even made unsuccessful suicide attempts.

Although the implementation of such ineffective passive approaches sometimes helped NF1 patients achieve relative peace, this feeling of peace and calmness was so fragile that it was disrupted by the persisting complications of the condition and its negative effects. As a result, the feeling of failure experienced was intensified in NF1 patients. Over time, NF1 patients lost their ability to tolerate the disease; thus, they considered other approaches that they believed to be more effective in helping them achieve peace in life. Thus, they started contemplating suicide as a way to escape the disease and to achieve eternal peace by ending their lives.

**Figure 1:**
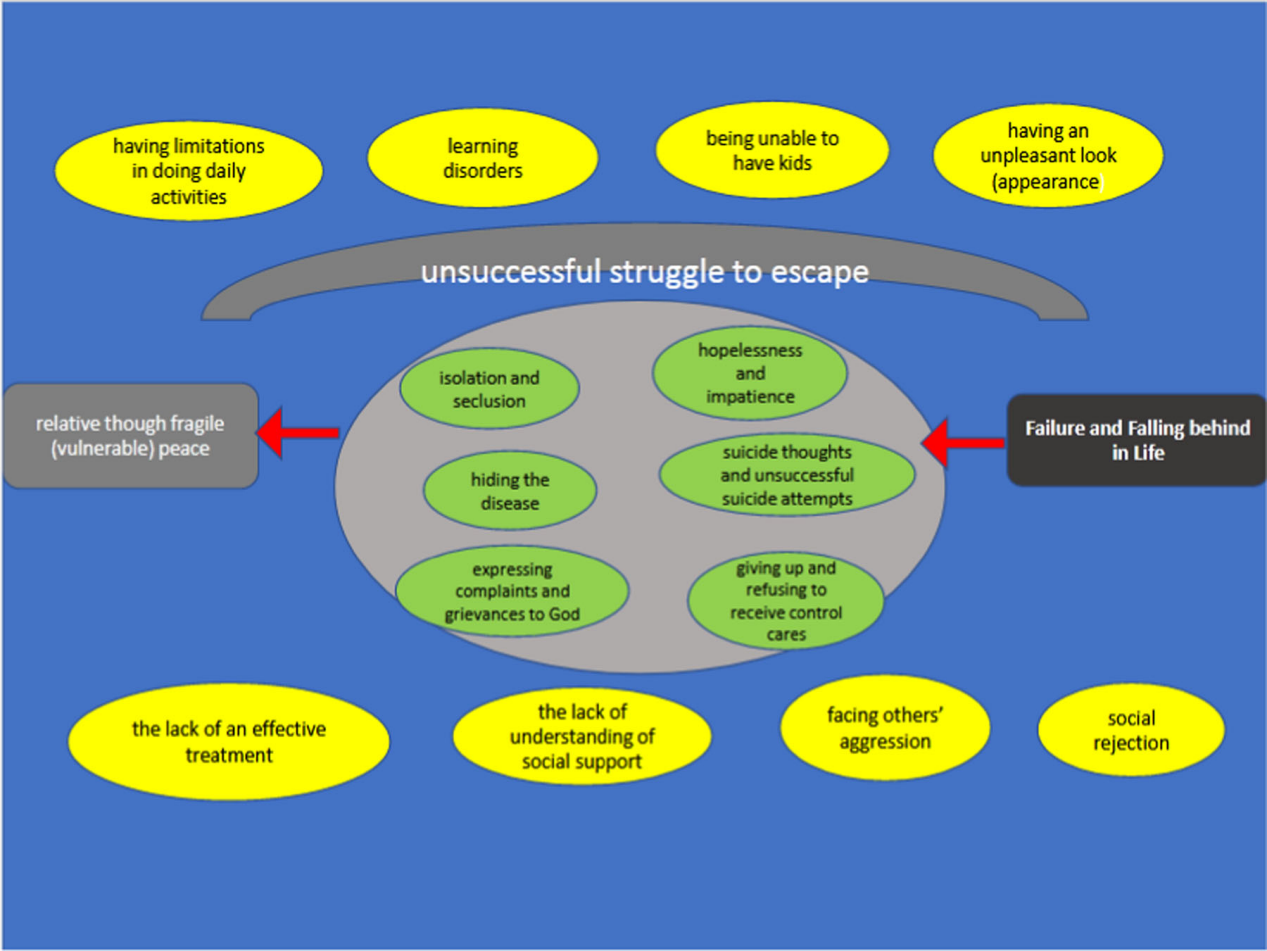
Theoretical statement: ‘unsuccessful struggle to escape’ as the approach that participants implemented to deal with neurofibromatosis

Therefore, this process of action and reaction, from the feelings of failure in life, to a relatively fragile sense of peace, was indicative of the ‘unsuccessful struggle to escape’ in response to the feelings of failure and falling behind in life (Figure 1).

## 4 DISCUSSION

The primary purpose of the present study was to explore the experiences of patients living with NF1. Data analysis revealed that the life of NF1 patients and their response to the feelings of failure and falling behind in life can be best described as ‘an unsuccessful struggle to escape’.

Feelings of failure and falling behind in life were determined to be the main concern and worry among NF1 patients. These feelings contributed towards failure in marriage, employment and school achievement. The findings of another similar study indicated that as NF1 patients have visible complications and symptoms, they face discrimination in terms of employment. The findings of another similar study indicated that as NF1 patients have visible cutaneous nodules, they deal with job discrimination; apparent disfigurement negatively affects the employee's ability to obtain or maintain employment.^14^ Beauty and physical health can increase a person's chances of getting hired and getting a job.^15^ Failure in marriage and the visibility of complications and symptoms have a negative influence on NF1 patients' ability to start a relationship. In this respect, the male NF1 patients in this study reported that they faced serious challenges in finding a life partner.^16^

The effective contextual factors that led to feelings of failure and falling behind in life among NF1 patients included unusual appearance due to spots, patches and tumours all over the body, inability to have children due to the risk of genetic transferability of the disease, learning disabilities, limitations in performing daily activities, social rejection and isolation, facing aggression from others, perception of no social support and incurability of the disease. The findings of the present study were in line with the results of the study carried out by Crawford et al.^2^, ^17^ in Australia on the potential effects of NF1 on the life and health of adults. They found that five main factors influence the life of the patients: (1) the unusual appearance of the patient, (2) learning disabilities and problems, (3) concern over having a child with the disease, (4) unknown progression of the disease and (5) the pain experienced. Three of these five factors were similar to the factors identified in the present study.^17^

Learning disability was another factor that affected feelings of failure and falling behind in life among NF1 patients. Along with other contextual factors, learning disabilities among NF1 patients can lead to feelings of failure and falling behind in life. Learning disabilities can lead to failure at school. Granstrom et al.^18^ showed that NF1 patients had painful memories from school. The participants in his study reported feelings of depression and dissatisfaction, and being labelled as lazy by their teachers.

Genetic transferability was another factor, along with other factors, that led to feelings of failure and falling behind in life among NF1 patients. In a study conducted by Farhi et al.,^19^ it was found that NF1 couples who planned to have children underwent diagnostic interventions before the birth of their children so that It was found that NF1 couples who intended to have a child, requested for the prenatal diagnostic procedures, with the aim of preventing the passing of the defective gene to the child. Moreover, it was observed that none of the couples were willing to have more children again without medical interventions.^19^

The participants in the present study considered neurofibromatosis as a limiting condition. In a systematic qualitative review, Von de Lippe et al.^20^ studied patients with rare diseases, including scleroderma, haemophilia, Phenylketonuria and Wilson's disease, and showed three important themes in the lives of these patients: (1) consequences of having a life with a rare disorder, (2) social aspects of a life with a rare disorder and (3) patient experiences with the health care system.^20^

In this respect, the study by Dalgard et al.^21^ on the mental stress caused by skin disorders suggested that aggression, social rejection and other social problems may intensify negative feelings, incompatible thought processes (the feeling of deficit), negative self‐perception (reduced self‐esteem and having a negative physical image of oneself) and negative behavioural patterns (too much social isolation). It is not a coincidence that sociopsychological complications such as depression, suicidal thoughts and anxiety are widespread among these patients.^21^

## 5 CONCLUSIONS

The findings of the present study indicated that although neurofibromatosis is mainly a physical genetic disorder, patients suffering from this disease face painful emotional and social experiences and suffering due to the cosmetic deformities caused by the disease. Currently, it is impossible for the majority of NF1 patients to undergo cosmetic surgeries. Hence, it is advisable to provide psychiatric and psychological support to these patients. In this way, the patients may learn coping skills that can help them cope with the mental suffering that they experience.

## 7 CONFLICT OF INTERESTS

The authors declare that there are no conflict of interests.

## ETHICS STATEMENT

All important ethical considerations were followed during the course of the present study, including obtaining formal permits from the authorities, introducing researchers to the participants, informing the participants about the objectives of the study and the length of interviews, obtaining formal written consent from all participants before the start of the study, obtaining permission to record interviews from the participants, letting the participants know that they could leave the study at any stage at their own volition and assuring the participants of the anonymity of their personal information and confidentiality of their data.

## Data Availability

The data that support the findings of this study are available on request from the corresponding author. The data are not publicly available due to privacy or ethical restrictions.
